# Personal victimization experiences of autistic and non-autistic children

**DOI:** 10.1186/s13229-022-00531-4

**Published:** 2022-12-24

**Authors:** Natalie Libster, Azia Knox, Selin Engin, Daniel Geschwind, Julia Parish-Morris, Connie Kasari

**Affiliations:** 1 Department of Education, UCLA, 457 Portola Plaza, Los Angeles, CA 90024 USA; 2 Center for Autism Research and Treatment, Semel Institute for Neuroscience, UCLA, 760 Westwood Plaza, Los Angeles, CA 90024 USA; 3 Children’s Hospital of Philadelphia, Center for Autism Research, 2716 South St, Philadelphia, PA 19104 USA; 4 David Geffen School of Medicine, UCLA, 10833 Le Conte Ave, Los Angeles, CA 90024 USA; 5 Department of Psychology, University of Pennsylvania, 3720 Walnut St, Philadelphia, PA 19104 USA

**Keywords:** Autism spectrum disorder, Bullying victimization, Autism symptom severity, Social affect, Sex differences

## Abstract

**Background:**

Autistic children report higher levels of bullying victimization than their non-autistic peers. However, autistic children with fewer social difficulties, as measured on the Autism Diagnostic Observation Schedule (ADOS), are more likely to report being bullied. Autistic children with stronger social skills may not only be more likely to identify and report incidents of bullying, but they may also be more likely to interact with their non-autistic peers, increasing their likelihood of being victimized. Autistic girls may be especially at-risk of experiencing bullying victimization, as a growing body of research suggests that autistic girls demonstrate fewer social difficulties and are more socially motivated than autistic boys. Here, we explored reported problems with peers and bullying victimization among a carefully matched sample of autistic and non-autistic boys and girls. Qualitative methods were further implemented to gain a more holistic understanding of the social experiences of autistic boys and girls.

**Methods:**

This mixed-methods study analyzed the transcribed clinical evaluations of 58 autistic children (29 girls) matched to 42 non-autistic children (21 girls) on age and IQ. Within each diagnostic group, boys and girls were matched on ADOS severity score. We compared reported problems with peers and bullying victimization across sex and diagnosis. Among autistic children, we further examined whether ADOS social affect (SA), restricted repetitive behaviors, and severity scores predicted problems with peers and bullying victimization. We then identified themes related to personal experiences of victimization.

**Results:**

Autistic children were more likely than non-autistic children to have experienced bullying victimization, and autistic children with lower ADOS severity and SA scores were more likely to report having been bullied. While autistic boys and girls reported similar levels of bullying victimization, qualitative analyses revealed sex differences in the underlying causes of peer conflict.

**Limitations:**

This study was a secondary data analysis. The standardized set of questions on the ADOS limited the amount of information that children provided about their peer relationships, and variations in follow-up questions may have influenced children’s responses.

**Conclusions:**

Although autism symptomatology places autistic children at greater risk for bullying victimization compared to their non-autistic peers, greater social challenges among autistic children are associated with lower rates of victimization. This study further highlights the importance of using mixed-methods approaches to discover nuances in the social experiences of autistic girls and boys that may become opportunities for support.

## Introduction

Autistic students experience higher rates of bullying victimization compared to neurotypical students and those with other special education needs [1–4]. Several characteristics associated with autism may put autistic children at-risk for bullying victimization. Autism spectrum disorder (ASD) is a neurodevelopmental condition marked by restricted interests, communication challenges, and difficulties with peer relationships [5]. Having friends and social support has been shown to be a protective factor for bullying victimization [6–8]. However, autistic children experience difficulties with developing and maintaining friendships, increasing their likelihood of becoming a target for bullying [4, 9, 10]. Reduced cognitive empathy—the ability to understand others’ perspectives—is another feature of autism that may contribute to autistic children’s increased likelihood of being bullied [4, 11]. Challenges with cognitive empathy make it difficult for autistic children to interpret social cues and take others’ perspectives. Therefore, when autistic children receive social feedback from their non-autistic peers, they may struggle to change their behavior to align with group norms [4]. Autistic children who are considered “different” by failing to align with social norms are likely to be victimized by their non-autistic peers [12]. However, autistic children have been shown to demonstrate heightened emotional empathy—the ability to directly feel others’ emotions [13]. In contrast, non-autistic children who perpetrate bullying exhibit reduced emotional empathy [14, 15]. Reduced empathy among non-autistic bullies and autistic victims supports Milton’s [16] “double empathy problem,” which implies that both non-autistic and autistic individuals have equal difficulty empathizing with one another [16].

### Methodological inconsistencies in the bullying literature

Prevalence estimates of bullying victimization among autistic children have varied due to methodological variations across studies, with rates ranging from 34 to 54% [17]. Prior studies have examined bullying victimization using various methods, informants, assessment time frames, and definitions of bullying [4]. Parent- and self-reports of bullying victimization have been shown to be inconsistent [18, 19]. Since parents only observe their children’s social interactions in specific contexts, they may naturally have different perceptions of their children’s victimization experiences. However, it is unclear exactly how parents’ perceptions differ from those of their children. Some parents report higher rates of victimization than their children [18], whereas some children report higher rates of victimization than their parents [19]. Nonetheless, self-reports, not parent-reports, of bullying victimization in autistic children have been shown to be associated with internalizing symptoms [18], suggesting the negative outcomes associated with bullying victimization are related to children’s own perceptions of being victimized. Variations in measures of bullying victimization and in measures of autism symptomatology may further contribute to inconsistent results across studies. For example, autistic children with greater social difficulties as measured on the Social Responsiveness Scale (SRS-P) [20] report more frequent verbal and social victimization (as measured on a modification of the Schwartz Peer Victimization Scale [21]) [18], but do not report more frequent overt, relational, or reputational victimization (as measured on the Revised Peer Experiences Questionnaire [22]) [23]. Meanwhile, autistic children with greater social difficulties as measured on the Autism Diagnostic Observation Schedule (ADOS-2) [24] report lower levels of physical and verbal victimization during semi-structured interviews [1].

Although the SRS-P and ADOS both measure autism symptomatology, the correlation between these two assessments is weak, potentially due to the different contexts in which the assessments are administered [25, 26]. The ADOS is administered by clinicians in schools and research settings, while the SRS-P is a parent-report of children’s behaviors at home. Moderate correlations have been found between teacher- and clinician-report measures, suggesting that perhaps autistic children behave similarly in school and research settings, which are relatively structured, but exhibit different behaviors in the unstructured settings of their homes [25, 26]. Teachers and clinicians, who observe autistic children in relation to other children, may also have different perspectives than parents, who may only focus on their child’s behavior [25]. Therefore, compared to the SRS-P, ADOS scores may better reflect children’s social behaviors in school and in other structured environments [25].

### Variability in bullying victimization among autistic children

Although characteristics associated with autism place autistic children at greater risk for bullying victimization compared to their non-autistic peers [4], autistic children with greater social difficulties—as measured on the ADOS—are less likely to report being bullied [1]. Variability in bullying victimization among autistic children may be attributed to differences in social awareness, motivation, environment, and sex. Autistic children with fewer social difficulties may be more aware of bullying behaviors, particularly subtle forms of aggression, and therefore report more instances of bullying victimization [1]. Autistic children with fewer social difficulties may also be more motivated to interact with their peers, increasing their likelihood of being teased or bullied, whereas autistic children who avoid interacting with their peers may evade being targeted [1]. Furthermore, autistic children’s reported victimization may depend on their peer environment. Bullying behaviors may be less likely to occur in specialized classrooms that have more teachers and support staff than mainstream classrooms, where adult supervision is limited [1]. This explanation was proposed by Rowley et al. [1], who found that autistic children with greater social difficulties in mainstream and specialized schools did not report differences in victimization, whereas autistic children with fewer social difficulties experienced higher levels of victimization in mainstream classrooms.

Autistic boys and girls have also been shown to have qualitatively different social experiences. Compared to autistic boys, autistic girls have more success maintaining reciprocal interactions and initiating friendships [27]. Autistic girls are also more likely to stay in close proximity to their peers during free play, optimizing their social opportunities, whereas autistic boys tend to play alone away from their peers [28]. However, the literature on bullying victimization among autistic children has focused on males, which is expected given the male-to-female ratio of autism being 4:1 [5]. Girls are underdiagnosed and remain under-represented in research [29]. Few studies have explored sex differences in peer conflict among autistic children, and methodological variations across these studies limit the conclusions that can be drawn. For example, a study by Sedgewick et al. [30] found that in mainstream classrooms, autistic girls reported higher rates of overall victimization and relational victimization than autistic boys and neurotypical girls and boys. However, another study by Sedgewick et al. [31] found that in specialized classrooms, autistic boys and girls reported significantly lower levels of conflict compared to their non-autistic classmates—though during semi-structured interviews, autistic girls revealed more instances of relational aggression than boys, including gossiping, exclusion, and “stealing” friends. Inconsistencies in these findings could be attributed to varying autism symptom severity across samples, and also to widely different peer contexts. As demonstrated by Rowley et al. [1], autistic children, especially those with stronger social skills, may be more likely to experience bullying in mainstream classrooms, in part because their “almost socially good enough” behavior elicits stronger negative reactions from non-autistic peers than from peers that have other special education needs. Furthermore, non-autistic girls tend to engage in more relational aggression than non-autistic boys, while non-autistic boys tend to engage in more direct aggression [32]. Therefore, autistic girls in mainstream classrooms may be particularly at-risk of experiencing relational aggression and being excluded by their peers.

### The current study

The current mixed-methods study had three aims. Based on direct interviewing of children, we aimed to determine if autistic children would report higher levels of peer conflict and bullying victimization compared to matched non-autistic children on age, sex, IQ, and severity of autism symptomatology. While prior studies [30, 31] matched boys and girls in the autistic and control samples on age and IQ, they did not match the boys and girls in each group on autism symptom severity. The current study matched autistic boys and girls, as well as non-autistic boys and girls, on ADOS severity scores to control for autism symptom severity when examining sex differences in peer victimization. We also aimed to determine if autism symptom severity, social affect (SA), and restricted repetitive behaviors (RRB) as measured on the ADOS predicted problems with peers and bullying victimization among autistic children. Finally, we aimed to determine if there were qualitatively different social experiences of autistic boys and girls as reported from observational studies at school [27, 28].

## Methods

### Participants

The current mixed-methods study was a secondary analysis of transcribed clinical evaluations from 100 children between 6 and 15 years—58 autistic children (29 girls) and 42 non-autistic children (21 girls). The transcribed evaluations were selected from two ongoing studies and five completed studies in which participants were administered the ADOS (Module 3) [24]. The two ongoing studies (P50HD055784, 2R01MH100027-11) are currently being conducted at the University of California, Los Angeles. Two of the completed studies [33, 34] took place at UCLA, one study [35] took place at both the University of North Carolina, Chapel Hill and the University of Chicago, and the other two studies [36, 37] took place at Children’s Hospital of Philadelphia. The number of participants whose data was used from each study is listed in Table 1. The UCLA Institutional Review Board (IRB) approved data sharing of all seven primary studies, as did the IRB at each site.

**Table 1:** Primary studies from which current sample was selected

	ASD boys	ASD girls	Non-ASD boys	Non-ASD girls
*Ongoing studies*				
P50HD055784	2	2	–	–
2R01MH100027-11	4	4	–	–
*Completed studies*				
Dean et al. (2020)	1	1	–	–
Kasari et al. (2016)	2	3	–	–
Lord et al. (2006)	1	1	–	–
Cola et al. (2022)	10	8	17	14
Parish-Morris et al. (2017)	9	10	4	7
Total	29	29	21	21

This study focused on autistic children who were administered Module 3 of the ADOS. Thus, they were children with spoken language (including complex sentences and references to non-present people or events) and who had average to above average IQs. The transcribed evaluations of non-autistic children were selected from the two studies at Children’s Hospital of Philadelphia [36, 37], in which semi-structured behavioral samples of non-autistic children were compared to those of autistic children. In the current study, autistic and non-autistic boys and girls were matched by group (frequency matching) on age (range: 6–15 years) and IQ. Two one-way ANOVAs were conducted to test the success of the matching procedure and did not reveal significant differences in age and IQ across the four sex/diagnostic groups (see Table 2). Furthermore, ADOS calibrated severity scores (CSS) were matched across autistic boys and girls and across non-autistic boys and girls. Independent samples t tests were conducted to test the success of the matching procedure and did not reveal significant differences in ADOS severity scores across sex within each diagnostic group. ADOS social affect (SA) and restricted repetitive behaviors (RRB) scores also did not differ across sex within each diagnostic group, as tested using independent samples t tests (see Table 2).

**Table 2:** Participant characteristics by sex and diagnostic group

	Age	IQ	ADOS CSS	ADOS SA	ADOS RRB
	*M* (*SD*)	*M* (*SD*)	*M* (*SD*)	*M* (*SD*)	*M* (*SD*)
ASD boys (*n* = 29)	10.48 (1.64)	104.14 (14.90)	6.45 (2.25)	6.69 (2.17)	6.14 (3.07)
ASD girls (*n* = 29)	10.41 (1.97)	103.69 (17.09)	6.31 (2.44)	6.34 (2.33)	6.83 (2.22)
*M *(*SD*)	10.45 (1.80)	103.91 (15.90)	6.38 (2.32)	6.52 (2.24)	6.48 (2.68)
*p*	0.89	0.92	0.82	0.56	0.33
Non-ASD boys (*n* = 21)	9.90 (2.64)	108.29 (10.83)	1.19 (0.40)	1.86 (0.91)	1.67 (1.71)
Non-ASD girls (*n* = 21)	9.81 (2.71)	105.14 (14.91)	1.29 (0.78)	1.81 (1.17)	1.67 (1.71)
*M *(*SD*)	9.86 (2.65)	106.71 (12.97)	1.24 (0.62)	1.83 (1.03)	1.67 (1.69)
*p*	0.91	0.44	0.62	0.88	0.99
Total *M *(*SD*)	10.20 (2.20)	105.09 (14.73)	–	–	–
*p*	0.62	0.72	–	–	–

Racial and ethnic demographic information was available for 99 of the 100 participants—68% of participants were White, 19% were Black, 3% were Hispanic, 3% were Asian, and 6% were multiracial. Participants had been administered the ADOS and demonstrated evidence that they understood the assessment questions and could articulate their responses. Children who did not respond to at least 50% of the ADOS interview questions were excluded from the study, as rich and comprehensive qualitative data were needed to conduct the thematic analysis.

### Measures

In the seven primary studies, the ADOS (Module 3) [24] was administered in a range of settings—clinicians either visited the child’s school or home or the child visited the institution where the study was taking place. Each administration of the ADOS was videotaped for later analysis. The ADOS is a semi-structured diagnostic assessment used to measure behaviors that may be symptomatic of ASD, including challenges in communication, social interaction, and play. After the assessment is finished, the clinician rates a series of items based on the child’s performance and observations made during the assessment [38]. These ratings are used to formulate diagnostic algorithms for two behavioral domains—social affect (SA) and restricted repetitive behaviors (RRB). The SA and RRB algorithms are then totaled and standardized to provide a measure of overall autism symptom severity, known as the calibrated severity score (CSS) [39, 40]. Calibrated severity scores can be used to compare autism symptom severity across children of different ages [40]. For the purposes of this study, participants’ SA and RRB raw scores were also standardized to provide calibrated domain scores. Calibrated severity and domain scores, which range from 1 (least severe) to 10 (most severe), are less influenced by non-autism child characteristics, such as language skills and age, than raw totals [39, 40].

In the current study, children’s responses to interview questions on the ADOS (Module 3) were coded, with a focus on questions about whether children experienced difficulties getting along with people at school and whether they had ever been teased or bullied. Unlike Rowley et al. [1], who coded children’s responses to these questions into one of five categories, the current study quantitatively analyzed children’s responses to both questions, thereby exploring subtle conflict with peers and overt bullying victimization. Furthermore, an inductive thematic analysis was conducted to identify sex differences in children’s personal victimization experiences. The qualitative methods implemented in this study allowed for a more holistic understanding of children’s perceived experiences with peer conflict and bullying victimization.

#### Problems with peers and bullying victimization (quantitative)

For the purposes of this study, children were asked (1) “Have you ever had problems getting along with people at school?” and (2) “Have you ever been teased or bullied?” Responses were transcribed and coded for problems with peers and bullying victimization. “Yes” was coded if the child reported having experienced problems with peers and “no” was coded if the child did not report having experienced problems with peers. Similarly, “yes” was coded if the child had experienced bullying and “no” was coded if the child had not experienced bullying. Responses that were coded as “yes” for bullying victimization met the definition provided by the American Psychological Association: “A form of aggressive behavior in which someone intentionally and repeatedly causes another person injury or discomfort.” [41] Some children (*n* = 12) reported that they had been teased but not bullied, that they had been “playfully” teased, or they reported a teasing incident that did not seem malicious and/or repeated (e.g., “Teddy teased me for not having matching socks”). These responses were coded as “no” for bullying victimization. A research assistant applied the coding procedure to 40% of the transcripts to establish interrater reliability (Cohen’s kappa = 0.73).

Two separate logistic regression models were conducted to test whether sex and/or diagnosis predicted problems with peers and bullying victimization, after controlling for age and IQ. Another two logistic regression models were conducted to test whether ADOS severity scores of *autistic children* (*n* = 58) predicted problems with peers and bullying victimization, after controlling for sex, age, and IQ. Two final logistic regression models were conducted to test whether the calibrated SA and RRB domain scores of autistic children predicted problems with peers and bullying victimization, controlling for sex, age, and IQ. All six regression models were conducted using R version 4.1.0. While the interviews on the ADOS are semi-structured and allow for follow-up questions to children’s responses, clinicians follow a standardized interview protocol and receive standard training on its administration, along with reliability assessments. Most clinicians in the current study followed protocol—however, two interviews excluded the question on problems with peers and two excluded the question on bullying victimization. When this occurred, the child’s response was coded as “unclear.” Furthermore, some children did not clearly articulate whether or not they ever had problems with peers or whether they had experienced bullying. These responses were also coded as “unclear.” The “unclear” codes for problems with peers (*n* = 7) and bullying victimization (*n* = 13) were omitted from the analyses.

#### Personal victimization experiences (qualitative)

An inductive thematic analysis based on the guidelines established by Miles and Huberman [42] was conducted to identify themes related to personal experiences of victimization. While the questions on problems with peers and bullying victimization were *each* coded for the quantitative analysis, the qualitative analysis identified themes relating to children’s personal victimization experiences across all the interview questions. The interviews on social difficulties were first reviewed to gain an understanding of the data. Afterward, patterns or themes in the data were identified. A list of codes that represented these themes was then generated and applied to the text through detailed, line-by-line annotations of the transcripts. After the interviews on social difficulties were coded, the coding procedure was applied to the interviews on friends/loneliness. This was done to capture themes related to peer victimization that emerged when the child was discussing friendships and loneliness. The coding procedure was implemented using Dedoose software [43]. A second coder annotated 40% of the transcripts and interrater reliability was calculated (75%). Finally, overlapping themes and conceptual similarities in the data, as well as exceptions and differences, were identified [42].

## Results

### Problems with peers and bullying victimization

The first regression model tested predictors of problems with peers, in which the question “Have you ever had problems getting along with people at school?” was coded as “yes.” The second regression model tested predictors of bullying victimization, in which the question “Have you ever been teased or bullied?” was coded as “yes.” Since the interaction between sex and ASD diagnostic status was not a significant predictor of problems with peers or bullying victimization, this variable was taken out of the final two models, which are depicted in Table 3. Neither sex nor ASD diagnostic status predicted problems with peers—however, ASD diagnostic status, but not sex, significantly predicted bullying victimization. Autistic children were 7.77 times more likely than non-autistic children to have been bullied (*p* < 0.001). The transcripts of 48 autistic children who were asked the question, “Have you ever been teased or bullied?” were coded as “yes” or “no” (10 responses were coded as “unclear” and omitted from the analysis). Of these transcripts, 27 (58%) children reported having been bullied. Meanwhile, the transcripts of 39 non-autistic children who were asked the bullying question were coded as “yes” or “no” (3 children were unclear in their responses). Of these transcripts, only 6 (15%) children reported having been bullied.

**Table 3:** Predictors of problems with peers and bullying victimization among autistic and non-autistic children

Outcome	Predictor	OR	Lower OR	Upper OR	*p*
Problems with peers					
	Diagnosis	2.31	0.99	5.56	0.06
	Sex	1.28	0.55	3.02	0.56
	IQ	1.00	0.97	1.03	0.91
	Age	0.87	0.70	1.07	0.20
Bullying victimization					
	Diagnosis***	7.77	2.77	25.21	< 0.001
	Sex	0.47	0.17	1.25	0.13
	IQ	1.02	0.98	1.06	0.40
	Age	1.19	0.92	1.57	0.20

Asterisk indicates level of statistical significance: * *p* < 0.05, ** *p* < 0.01, *** *p* < 0.001

Another two logistic regression models tested whether ADOS calibrated severity scores (CSS) among autistic children predicted problems with peers and bullying victimization. The models are depicted in Table 4. The ADOS severity scores of autistic children did not significantly predict problems with peers, but did significantly predict bullying victimization. For every increase in ADOS CSS, autistic children were 0.72 times as likely to have been bullied (*p* = 0.04). Two final logistic regression models tested whether the calibrated SA and RRB domain scores of autistic children predicted problems with peers and bullying victimization. The models are depicted in Table 5. Neither SA nor RRB scores predicted problem with peers. However, SA scores, but not RRB scores, significantly predicted bullying victimization. For every increase in SA score, autistic children were 0.59 times as likely to have been bullied (*p* = 0.007).

**Table 4:** ADOS CSS as a predictor of problems with peers and bullying victimization among autistic children

Outcome	Predictor	OR	Lower OR	Upper OR	*p*
Problems with peers					
	ADOS CSS	0.94	0.73	1.20	0.63
	Sex	1.08	0.35	3.41	0.89
	IQ	0.99	0.95	1.03	0.63
	Age	1.10	0.77	1.60	0.61
Bullying victimization					
	ADOS CSS*	0.72	0.51	0.98	0.04
	Sex	0.84	0.24	3.05	0.79
	IQ	1.00	0.96	1.05	0.88
	Age	1.44	0.95	2.34	0.11

Asterisk indicates level of statistical significance: * *p* < 0.05, ** *p* < 0.01, *** *p* < 0.001

**Table 5:** ADOS SA and RRB as predictors of problems with peers and bullying victimization among autistic children

Outcome	Predictor	OR	Lower OR	Upper OR	*p*
Problems with peers					
	ADOS SA	0.81	0.60	1.07	0.15
	ADOS RRB	1.12	0.90	1.42	0.31
	Sex	0.96	0.29	3.13	0.94
	IQ	0.99	0.95	1.03	0.51
	Age	1.15	0.80	1.69	0.45
Bullying victimization					
	ADOS SA**	0.59	0.38	0.83	0.007
	ADOS RRB	1.20	0.92	1.60	0.20
	Sex	0.53	0.12	2.16	0.39
	IQ	1.00	0.95	1.05	0.93
	Age	1.57	1.01	2.66	0.06

Asterisk indicates level of statistical significance: * *p* < 0.05, ** *p* < 0.01, *** *p* < 0.001

### Personal victimization experiences

Two primary themes emerged from the data. These included (1) types of peer victimization, and (2) reasons for peer victimization. While autistic and non-autistic boys and girls reported similar types of peer victimization, there were sex differences in the underlying causes of peer conflict.

*Types of peer victimization* Codes for verbal, physical, and relational aggression were applied to the text when these themes emerged, even if the child claimed they had no problems with peers or had never been bullied. Verbal aggression included instances in which the child was called names or threatened by their peers:

Kids bullied and really teased me a lot and now I go to a better school…. they just said a lot of hurtful things to me. (Autistic boy, 10)

Physical aggression included instances in which the child was shoved, hit, kicked, punched, or otherwise physically violated:

So a lot of the boys in my class… they don't like me. I don't know why… they like throw stuff at us. (Non-autistic girl, 12)

Finally, relational aggression included instances in which the child’s relationships or social status was damaged (e.g., gossiping or exclusion):

They've been calling me names um…. haven't been so nice to me ever since I got in this school, and (Name)... I tried to be friends with him, he says no. (Autistic boy, 9)

There were no clear differences in reported instances of verbal, physical, or relational aggression between autistic and non-autistic children and between boys and girls. However, there were only 20 reported instances of verbal aggression (15 ASD, 14 boys), 13 reported instances of physical aggression (8 ASD, 6 boys), and 11 reported instances of relational aggression (7 ASD, 6 boys). All 13 instances of physical aggression reported by participants were perpetrated by boys.

*Reasons for peer victimization* Children often gave explanations for why they had trouble getting along with peers or why they were bullied. Certain explanations were expressed by both autistic and non-autistic children and by both boys and girls. For example, autistic and non-autistic boys and girls reported social difficulties because of their personalities or because they were considered different from their peers:

The beginning of the year… I wasn’t so nice to kids, like terrible… like I was mean. (Non-autistic girl, 6)

I actually have a lot of friends but most people they don’t like me… because they think I’m weird. (Autistic boy, 10)

Another reason for peer victimization—expressed by autistic and non-autistic boys—was jealousy toward their own positive attributes:

Well um there's some people who are just really annoyed with me sometimes... um sometimes because um as far as I can tell they get annoyed that I'm smarter than them. (Non-autistic boy, 11)

They're jealous of how good I'm doing and they want to make… and they want to make themself feel better. (Non-autistic boy, 9)

Meanwhile, other explanations were only expressed by autistic children. For example, several autistic girls reported social difficulties because they had trouble being flexible or became easily upset:

If they choose a game that I don't want to play, I kind of get mad at them… I just tell them that I don't want to play it and normally they just say, well we're going to play it without you if you don't want to play it. And I get really mad. (Autistic girl, 9)

Sometimes I get upset easily… I try to calm down though… I need to try harder. (Autistic girl, 13)

A final explanation expressed by autistic boys was difficulty with social interactions.

Sometimes I play with them in a way that they don’t like… I don’t really rely on personal space when I play with stuff. (Autistic boy, 9)

I try to become their friend so much that I become a little bit creepy…. not creepy… just weird. (Autistic boy, 9)

Therefore, while autistic boys and girls were equally likely to be victimized, the reasons why they experienced peer conflict differed. Autistic boys demonstrated social difficulties that affected their ability to play and socialize with their peers. While autistic girls did not report having these social challenges, they often encountered difficulties with cognitive flexibility and emotion control, which affected their relationships with their peers.

## Discussion

This study aimed to understand the extent to which boys and girls were similar or different on self-reports of peer relationships and peer victimization. The mixed methods used in this study yielded three main findings. First, ASD diagnostic status was a significant predictor of active bullying victimization, but not of milder problems with peers. Autistic children were significantly more likely than non-autistic children to report having been bullied, but were not more likely to report having had problems with peers. A possible explanation for this discrepancy is that autistic children may have difficulty perceiving subtle problems with peers, which can be less discernable than being bullied. This difficulty was noted in a prior study [17] in which the researchers found that autistic children struggled to conceptually understand certain items pertaining to relational victimization on the Schwartz Peer Victimization Scale [20]. Similarly, autistic boys and girls in specialized classrooms have been shown to report significantly lower levels of conflict compared to their non-autistic classmates in a rating scale format—however, during semi-structured interviews, autistic girls reveal instances of relational aggression within their friendships [31]. This suggests that autistic children, especially girls, may not always report acts of relational aggression as instances of peer conflict per se.

The second main finding addressed whether autism symptom severity, including challenges with social affect and restricted repetitive behaviors, as measured on the ADOS predicted problems with peers and bullying victimization among autistic children. Autistic children with *lower* ADOS severity scores and SA domain scores were significantly *more* likely to have been bullied, supporting the findings of Rowley et al. [1] Autistic children with fewer social challenges may be more aware of bullying behaviors and therefore report more instances of bullying victimization. Furthermore, autistic children with fewer social challenges may be more motivated to interact with their peers, increasing their likelihood of being a target for bullying. Meanwhile, we did not find sex to be a significant predictor of problems with peers or bullying victimization among autistic and non-autistic children. Contrary to the findings of Sedgewick et al. [30], boys and girls in the current study were as likely to report having had problems with peers and having been bullied. It is important to note that in Sedgewick et al.’s study [30], autistic boys had significantly higher ADOS severity scores than autistic girls (*M* = 5.45, *SD* = 1.90 vs. *M* = 3.87, *SD* = 1.89). If autism symptom severity predicts reported bullying victimization, as demonstrated in the current study, lower ADOS severity scores among autistic girls versus autistic boys in Sedgewick et al.’s study [30] may have led these children to report higher rates of peer conflict. In the current study, we matched participants on ASD symptom severity and found no significant difference in ADOS severity scores between autistic boys (*M* = 6.45, *SD* = 2.25) and girls (*M* = 6.31, *SD* = 2.44). This may explain why autistic boys and girls in our sample reported similar levels of bullying victimization.

The third main finding was the qualitative differences in the social experiences of autistic boys and girls. Autistic boys often experienced social difficulties because they had trouble interacting with their peers; for example, they would talk too much in conversations or disregard personal space. These social challenges could underlie observations that autistic boys tend to play alone away from their peers on the playground [28]. Although there were no significant differences in ADOS SA scores between autistic boys and girls in the current study, autistic girls did not report having these social challenges. This may be due to the tendency of autistic girls to copy the social behaviors of their non-autistic peers, thereby “masking” their social difficulties [28]. Several studies report that the *apparent* social success of autistic girls is due to their ability engage in masking behaviors that conceal potential communication challenges [27, 28, 44]. However, autistic girls in the current study still experienced peer conflict, often because they became easily upset or had trouble with flexibility; for example, they would insist on playing a game they liked even if their peers wanted to play something else. This finding supports prior research, demonstrating that autistic girls exhibit greater challenges with parent-reported executive functioning than autistic boys [45]. Executive functioning (EF) consists of behaviors that are necessary for goal-directed and intentional problem-solving, including cognitive flexibility and emotion control [46]. These behaviors are particularly difficult for autistic children [47, 48], especially autistic girls [45]. Since EF was not measured in the current study, it is possible that autistic boys and girls in the sample found these behaviors equally challenging. However, difficulties with EF only seemed to affect the peer relationships of autistic girls.

### Limitations

This study has many strengths, including a relatively large, well-matched sample of autistic and non-autistic girls and boys. It also has limitations, which we hope will be addressed in future research. The ADOS is a standardized measure, but there was still variability in the administration of the assessment across participants. There were variations in obtaining answers to all of the questions, and follow-up questions asked by clinicians may have influenced children’s responses—clinicians who asked more follow-up questions may have elicited more information from children. Furthermore, the current study only included participants who responded to at least 50% of the interview questions. While this was necessary to collect rich qualitative data, we were unable to examine peer conflict in autistic children who did not have the verbal communication skills to talk about their experiences. Autistic children with greater communication difficulties may be bullied to the same extent as those with fewer difficulties but may struggle to report these incidents. Future studies should therefore use multi-informant approaches, including self-, parent-, and teacher-report, to acquire a more holistic understanding of autistic children’s social experiences in school contexts. The generalizability of the current study is also limited by race, as the majority of the participants were White.

A strength of the current study was the wide age range of the sample, which spanned from early childhood (6 years) through adolescence (15 years). While there were no clear differences in peer victimization across development, prior research has found victimization to be most prevalent during middle childhood (11–13 years of age) compared to other ages [49–51]. Since the majority of participants in the current study were between 9 and 12 years of age (55%), there may not have been enough children in early childhood and adolescence to capture differences in peer victimization across development. Future studies should therefore include larger samples of children across different developmental stages. Since younger children may have more difficulty identifying acts of bullying and consequently underreport incidents of peer conflict [52], these studies should also use multi-informant approaches as previously discussed. Other risk and protective factors of bullying victimization may have also been unaccounted for in the current study. For example, co-occurring developmental conditions, such as ADHD, may have exacerbated problems with peers and bullying victimization but were not always clearly documented for each child. In addition, the type of schooling and the type of support that children received may have contributed to experienced victimization. For example, children who were homeschooled may have been protected from bullies, whereas children who received additional educational supports in mainstream school settings may have been more vulnerable to victimization.

Finally, it is important to note that the ADOS was developed with a predominantly male autistic population [24] and may not fully capture the presentation of autism in girls. Autistic girls with similar ADOS severity scores as autistic boys have been shown to exhibit greater autism severity as reported by parents on the SRS-P [53]. The autistic girls in Sedgewick et al.’s study [30] also scored significantly higher on the SRS-P than autistic boys, even though they demonstrated lower autism severity on the ADOS. Therefore, it is possible that the autistic girls in the current sample possessed more autistic traits than boys, resulting in similar severity scores on the ADOS. Furthermore, the ADOS does not measure EF skills, such as emotion control and cognitive flexibility, which are more challenging for autistic girls [45]. While many autistic girls in the current study described how difficulties with EF affected their relationships with peers, we were unable to measure EF across all the participants.

### Future directions and implications

Future directions stemming from this research can use multi-method approaches to explore sex differences in peer victimization among autistic and non-autistic children. For example, future studies could compare child, parent, and teacher reports as well as observational measures to examine discrepancies in perceived vs. observed conflict among autistic children. The current study also highlights the importance of using qualitative methods as well as quantitative methods in future research. Quantitative measures, such as yes/no or multiple-choice questionnaires, may not fully capture the social behaviors and experiences of autistic children. By asking autistic children questions about their relationships with peers, researchers may discover nuances in the social experiences of autistic girls and boys that become opportunities for support. Finally, actual real-time observations in context would enrich the data and interpretability of these findings.

The current study has important implications for parent- and school-mediated interventions designed to improve peer relationships in autistic children. First, it is essential that the parents of autistic children gain a deeper understanding of their children’s social experiences. Since autistic children may not readily perceive subtle problems with peers or relational forms of aggression, parents may need to take a more active role in understanding their children’s relationships with peers. With clinician support, parents can engage in discussions with their autistic children on how to identify exclusive behaviors and negative treatment by peers, as well as help them practice strategies to improve social success. On the other side of the social equation, school-based interventions can also be implemented to teach non-autistic children how to engage in positive interactions with children with social difficulties. Teachers can play a role in facilitating positive social interactions between autistic and non-autistic children, and also instruct their students how to model prosocial behaviors and interact with children with social difficulties at school.

## Conclusion

The current study demonstrated that autistic boys and girls were as likely to report experiencing subtle problems with peers as non-autistic boys and girls. However, autistic children were more likely to have been overtly bullied compared to non-autistic children. While one could assume that having fewer ASD symptoms would improve peer relationships, evidence suggests that stronger social skills may increase the likelihood of experiencing and reporting peer conflict. In addition, while autistic boys and girls reported similar levels of bullying victimization, qualitative analyses revealed sex differences in the underlying causes of peer conflict. The current study therefore highlights the importance of using mixed-methods approaches to understand perceived and experienced peer conflict among autistic children.

## Data Availability

The datasets generated and/or analyzed during the current study are not publicly available due to privacy concerns for minors with disabilities.

## Abbreviations

ADOS: Autism Diagnostic Observation Schedule–2nd edition
ASD: Autism spectrum disorder
CCS: Calibrated severity score
IQ: Intelligence quotient
M: Mean
OR: Odds ratio
RRB: Restricted repetitive behaviors
SA: Social affect
SRS-P: Social Responsiveness Scale-2nd edition (parent-report)
SD: Standard deviation

## References

[1] Rowley E, Chandler S, Baird G, Simonoff E, Pickles A, Loucas T, Charman T (2012). The experience of friendship, victimization and bullying in children with an autism spectrum disorder: associations with child characteristics and school placement. Res Autism Spectrum Disord.

[2] Symes W, Humphrey N (2010). Peer-group indicators of social inclusion among pupils with autistic spectrum disorders (ASD) in mainstream secondary schools: a comparative study. Sch Psychol Int.

[3] Twyman KA, Saylor CF, Saia D, Macias MM, Taylor LA (2010). Bullying and ostracism experiences in children with special health care needs. J Dev Behav Pediatr.

[4] Schroeder JH, Cappadocia MC, Bebko JM, Pepler DJ, Weiss JA (2014). Shedding light on a pervasive problem: a review of research on bullying experiences among children with autism spectrum disorders. J Autism Dev Disord.

[5] Maenner MJ, Shaw KA, Bakian AV, Bilder DA, Durkin MS, Esler A, Furnier SM, Hallas L, Hall-Lande J, Hudson A, Hughes MM (2021). Prevalence and characteristics of autism spectrum disorder among children aged 8 years—autism and developmental disabilities monitoring network, 11 sites, United States, 2018. MMWR Surveill Summ.

[6] Estell DB, Farmer TW, Irvin MJ, Crowther A, Akos P, Boudah DJ (2009). Students with exceptionalities and the peer group context of bullying and victimization in late elementary school. J Child Fam Stud.

[7] Hodges EVE, Perry DG (1999). Personal and interpersonal antecedents and consequences of victimization by peers. J Pers Soc Psychol.

[8] Martlew M, Hodson J (1991). Children with mild learning difficulties in an integrated and in a special school: comparisons of behaviour, teasing, and teachers’ attitudes. Br J Educ Psychol.

[9] Bauminger N, Kasari C (2000). Loneliness and friendship in high-functioning children with autism. Child Dev.

[10] Chamberlain B, Kasari C, Rotheram-Fuller E (2007). Involvement or isolation? The social networks of children with autism in regular classrooms. J Autism Dev Disord.

[11] Baron-Cohen S, Leslie AM, Frith U (1985). Does the autistic child have a “theory of mind”?. Cognition.

[12] Humphrey N, Lewis S (2008). ‘Make me normal’: the views and experiences of pupils on the autistic spectrum in mainstream secondary schools. Autism.

[13] Smith A (2009). The empathy imbalance hypothesis of autism: a theoretical approach to cognitive and emotional empathy in autistic development. Psychol Rec.

[14] van Noorden TH, Haselager GJ, Cillessen AH, Bukowski WM (2015). Empathy and involvement in bullying in children and adolescents: a systematic review. J Youth Adolesc.

[15] DeNigris D, Brooks PJ, Obeid R, Alarcon M, Shane-Simpson C, Gillespie-Lynch K (2018). Bullying and identity development: insights from autistic and non-autistic college students. J Autism Dev Disord.

[16] Milton DE (2012). On the ontological status of autism: the ‘double empathy problem’. Disabil Soc.

[17] Maiano C, Normand CL, Salvas MC, Moullec G, Aimé A (2016). Prevalence of school bullying among youth with autism spectrum disorders: a systematic review and meta-analysis. Autism Res.

[18] Adams RE, Fredstrom BK, Duncan AW, Holleb LJ, Bishop SL (2014). Using self-and parent-reports to test the association between peer victimization and internalizing symptoms in verbally fluent adolescents with ASD. J Autism Dev Disord.

[19] van Schalkwyk G, Smith IC, Silverman WK, Volkmar FR (2018). Brief report: bullying and anxiety in high-functioning adolescents with ASD. J Autism Dev Disord.

[20] Constantino JN, Gruber CP (2012). Social responsiveness scale: SRS-2.

[21] Schwartz D, Farver JM, Chang L, Lee-Shin Y (2002). Victimization in South Korean children’s peer groups. J Abnorm Child Psychol.

[22] Prinstein MJ, Boergers J, Vernberg EM (2001). Overt and relational aggression in adolescents: social-psychological adjustment of aggressors and victims. J Clin Child Psychol.

[23] Storch EA, Larson MJ, Ehrenreich-May J, Arnold EB, Jones AM, Renno P, Fujii C, Lewin AB, Mutch PJ, Murphy TK, Wood JJ (2012). Peer victimization in youth with autism spectrum disorders and co-occurring anxiety: relations with psychopathology and loneliness. J Dev Phys Disabil.

[24] Lord C, Rutter M, DiLavore PC, Risi S, Gotham K, Bishop S (2012). Autism diagnostic observation schedule.

[25] Duvekot J, van der Ende J, Verhulst FC, Greaves-Lord K (2015). The screening accuracy of the parent and teacher-reported Social Responsiveness Scale (SRS): comparison with the 3Di and ADOS. J Autism Dev Disord.

[26] Reszka SS, Boyd BA, McBee M, Hume KA, Odom SL (2014). Brief report: concurrent validity of autism symptom severity measures. J Autism Dev Disord.

[27] Hiller RM, Young RL, Weber N (2014). Sex differences in autism spectrum disorder based on DSM-5 criteria: evidence from clinician and teacher reporting. J Abnorm Child Psychol.

[28] Dean M, Harwood R, Kasari C (2017). The art of camouflage: gender differences in the social behaviors of girls and boys with autism spectrum disorder. Autism.

[29] Humphrey N, Hebron J (2015). Bullying of children and adolescents with autism spectrum conditions: a ‘state of the field’ review. Int J Incl Educ.

[30] Sedgewick F, Hill V, Pellicano E (2019). ‘It’s different for girls’: gender differences in the friendships and conflict of autistic and neurotypical adolescents. Autism.

[31] Sedgewick F, Hill V, Yates R, Pickering L, Pellicano E (2016). Gender differences in the social motivation and friendship experiences of autistic and non-autistic adolescents. J Autism Dev Disord.

[32] Card NA, Stucky BD, Sawalani GM, Little TD (2008). Direct and indirect aggression during childhood and adolescence: a meta-analytic review of gender differences, intercorrelations, and relations to maladjustment. Child Dev.

[33] Kasari C, Dean M, Kretzmann M, Shih W, Orlich F, Whitney R, Landa R, Lord C, King B (2016). Children with autism spectrum disorder and social skills groups at school: a randomized trial comparing intervention approach and peer composition. J Child Psychol Psychiatry.

[34] Dean M, Williams J, Orlich F, Kasari C (2020). Adolescents with autism spectrum disorder and social skills groups at school: a randomized trial comparing intervention environment and peer composition. Sch Psychol Rev.

[35] Lord C, Risi S, DiLavore PS, Shulman C, Thurm A, Pickles A (2006). Autism from 2 to 9 years of age. Arch Gen Psychiatry.

[36] Parish-Morris J, Liberman MY, Cieri C, Herrington JD, Yerys BE, Bateman L, Donaher J, Ferguson E, Pandey J, Schultz RT (2017). Linguistic camouflage in girls with autism spectrum disorder. Mol Autism.

[37] Cola M, Yankowitz LD, Tena K, Russell A, Bateman L, Knox A, Plate S, Cubit LS, Zampella CJ, Pandey J, Schultz RT (2022). Friend matters: sex differences in social language during autism diagnostic interviews. Mol Autism.

[38] Lord C, Risi S, Lambrecht L, Cook EH, Leventhal BL, DiLavore PC, Pickles A, Rutter M (2000). The Autism Diagnostic Observation Schedule—Generic: a standard measure of social and communication deficits associated with the spectrum of autism. J Autism Dev Disord.

[39] Hus V, Gotham K, Lord C (2014). Standardizing ADOS domain scores: separating severity of social affect and restricted and repetitive behaviors. J Autism Dev Disord.

[40] Gotham K, Pickles A, Lord C (2009). Standardizing ADOS scores for a measure of severity in autism spectrum disorders. J Autism Dev Disord.

[41] American Psychological Association. (n.d.). Bullying. https://www.apa.org/topics/bullying

[42] Miles MB, Huberman AM (1994). Qualitative data analysis: an expanded sourcebook.

[43] Dedoose. Dedoose Version 8.0.35, web application for managing, analyzing, and presenting qualitative and mixed method research data. In: SocioCultural Research Consultants, LLC; 2018.

[44] Hull L, Petrides KV, Mandy W (2020). The female autism phenotype and camouflaging: a narrative review. Rev J Autism Dev Disord.

[45] White EI, Wallace GL, Bascom J, Armour AC, Register-Brown K, Popal HS, Ratto AB, Martin A, Kenworthy L (2017). Sex differences in parent-reported executive functioning and adaptive behavior in children and young adults with autism spectrum disorder. Autism Res.

[46] Gioia GA, Isquith PK, Kenworthy L, Barton RM (2002). Profiles of everyday executive function in acquired and developmental disorders. Child Neuropsychol.

[47] Hill EL (2004). Executive dysfunction in autism. Trends Cogn Sci.

[48] Kenworthy L, Yerys BE, Anthony LG, Wallace GL (2008). Understanding executive control in autism spectrum disorders in the lab and in the real world. Neuropsychol Rev.

[49] Brown SL, Birch DA, Kancherla V (2005). Bullying perspectives: experiences, attitudes, and recommendations of 9- to 13-year-olds attending health education centers in the United States. J Sch Health.

[50] Eslea M, Rees J (2001). At what age are children most likely to be bullied at school?. Aggress Behav Off J Int Soc Res Aggress.

[51] Peskin MF, Tortolero SR, Markham CM (2006). Bullying and victimization among Black and Hispanic adolescents. Adolescence.

[52] Levine E, Tamburrino M (2014). Bullying among young children: strategies for prevention. Early Childhood Educ J.

[53] Ratto AB, Kenworthy L, Yerys BE, Bascom J, Wieckowski AT, White SW, Wallace GL, Pugliese C, Schultz RT, Ollendick TH, Scarpa A (2018). What about the girls? Sex-based differences in autistic traits and adaptive skills. J Autism Dev Disord.

